# Sialylation as a checkpoint for inflammatory and complement-related retinal diseases

**DOI:** 10.3389/fncel.2025.1623755

**Published:** 2025-06-27

**Authors:** Yiduo Min, German Cuevas-Rios, Thomas Langmann, Harald Neumann

**Affiliations:** ^1^Institute of Reconstructive Neurobiology, Medical Faculty & University Hospital Bonn, University of Bonn, Bonn, Germany; ^2^Experimental Immunology of the Eye, Department of Ophthalmology, University Hospital Cologne, Cologne, Germany

**Keywords:** retina, sialylation, sialic acid, polysialic acid, complement, microglia, inflammation, age-related macular degeneration (AMD)

## Abstract

Sialylation is a modification process involving the addition of sialic acid residues to the termini of glycoproteins and glycolipids in mammalian cells. Sialylation serves as a crucial checkpoint inhibitor of the complement and immune systems, particularly within the central nervous system (CNS), including the retina. Complement factor H (FH), complement factor properdin (FP), and sialic acid-binding immunoglobulin-like lectin (SIGLEC) receptors of retinal mononuclear phagocytes are key players in regulating the complement and innate immune systems in the retina by recognizing sialic acid (Sia) residues. Intact retinal sialylation prevents any long-lasting and excessive complement or immune activation in the retina. However, sialylated glycolipids are reduced in the CNS with aging, potentially contributing to chronic inflammatory processes in the retina. Particularly, genetically induced hyposialylation in mice leads to age-related, complement factor C3-mediated retinal inflammation and bipolar cell loss. Notably, most of the gene transcript pathways enriched in the mouse retina, following genetically induced hyposialylation, are also involved in age-related macular degeneration (AMD). Interestingly, intravitreal application of polysialic acid (polySia) controlled the innate immune responses in the mouse retina by blocking mononuclear phagocyte reactivity, inhibiting complement activation, and protecting against vascular damage in two different humanized SIGLEC-11 animal models. Accordingly, a polySia polymer conjugate has entered clinical phase II/III testing in patients with geographic atrophy secondary to AMD. Thus, hyposialylation or dysfunctional sialylation should be considered as an age-related contributor to inflammatory retinal diseases, such as AMD. Consequently, sialic acid-based biologics could provide novel therapies for complement-related retinal diseases.

## 1 Introduction

The retina is an immune-privileged tissue with a blood–retinal barrier, composed of the microvascular endothelium and the retinal pigment epithelium (RPE) (Streilein et al., 2002; Chen et al., 2019), as well as an immune-suppressed microenvironment (Silverman and Wong, 2018; Murakami et al., 2020). However, genetic and environmental factors, such as diet and age, contribute to chronic immune responses that are associated with retinal degeneration (Amirul Islam et al., 2014; Thomas et al., 2021). In particular, age-related macular degeneration (AMD), a leading cause of blindness in individuals above 60 years, is closely associated with retinal inflammation and oxidative stress. AMD is classified into two forms: dry and wet (Mitchell et al., 2018). Dry AMD is characterized by the accumulation of drusen and the inability of the RPE to clear them. Wet AMD is characterized by blood–retinal barrier leakage, parenchymal edema, and choroidal neovascularization (CNV) driven by the vascular endothelial growth factor (VEGF) (Thomas et al., 2021; Marchesi et al., 2024). Notably, transcriptome analyses of retinas from AMD patients revealed upregulation of genes involved in the chemokine, cytokine, and complement cascade signaling pathways (Saddala et al., 2019).

In recent years, it has been found that sialylation, a post-translational modification of the glycocalyx termini by sialic acid residues (Schnaar et al., 2014), plays a key role in retinal complement and immune regulation. Sialic acid (Sia), the terminal carbohydrate of the glycocalyx, is found on mammalian cell surfaces in two types, N-acetylneuraminic acid (Neu5Ac) and N-glycolylneuraminic acid (Neu5Gc) (Varki et al., 2015; Pearce and Läubli, 2016). Only Neu5Ac is naturally present in humans due to a lineage-specific loss-of-function mutation in the gene encoding cytidine monophosphate-N-acetylneuraminic acid hydroxylase (CMAH) (Chou et al., 2002; Martin et al., 2005). Interestingly, several studies have shown that Neu5Gc from food sources, such as red meat, milk, and dairy products, can be incorporated into the glycocalyx of human tissue, potentially triggering immune responses against these non-human sialic acids, resulting in a condition termed xenosialitis (Oetke et al., 2001; Tangvoranuntakul et al., 2003; Hedlund et al., 2008). However, no evidence has been found of xenosialitis or Neu5Gc incorporation in the human retina. Moreover, Sias can form homopolymers with varying average degrees of polymerization (avDP) through glycosidic linkages. In mammals, polysialic acid (polySia) is a homopolymer composed of α2,8-linked Neu5Ac monomers, with a degree of polymerization ranging from 10 to approximately 100 (Schnaar et al., 2014). Sialylation can inhibit complement activation by targeting critical regulators of the complement cascade. In particular, complement factor H (FH) recognizes α2,3-linked Neu5Ac residues on the glycocalyx, thereby inhibiting the alternative complement pathway (Blaum et al., 2014). In addition, complement factor P (FP), a widely known positive regulator of the alternative complement pathway, has been shown to bind to low molecular weight α2,8-linked polySia with average degree of polymerization 20 (avDP20) and to reduce its ability to promote alternative complement activation. Sialylation also downregulates microglia activity by binding to sialic acid-binding immunoglobulin-like lectin (SIGLEC) receptors on immune cell membranes, serving as a checkpoint to maintain immune tissue homeostasis in the central nervous system (CNS; Klaus et al., 2021; Lünemann et al., 2021). However, intact sialylation is susceptible to aging and chronic inflammation. Thus, dysfunctional or reduced sialylation can disrupt retinal immune regulation, leading to excessive activation of the complement system, chronic inflammation, and tissue degeneration.

Given its essential role in retinal immune regulation, sialylation has become a promising therapeutic target for age-related and inflammatory retinal diseases (Nycholat et al., 2012; Rillahan et al., 2012; Zhong et al., 2022). Polysialylated ligands, such as polySia, have shown promising potential in preclinical studies to suppress complement activation, attenuate mononuclear phagocyte activity, and prevent vascular damage (Karlstetter et al., 2016; Krishnan et al., 2023).

This review explores the role of sialylation as a checkpoint in complement-associated retinal diseases, focusing on the mechanisms underlying dysfunctional sialylation, its impact on retinal immune homeostasis, and its therapeutic potential.

## 2 Dysfunctional sialylation during aging and chronic inflammation: a trigger for retinal diseases

Sialic acid is a nine-carbon sugar that forms the terminal cap of the glycol residues of glycolipids and glycoproteins. Sias of the glycocalyx contribute to various biological processes, such as microdomain formation (Möckl et al., 2015), cell adhesion (Kelm et al., 1994), tissue homeostasis (Varki and Gagneux, 2012), immune cell modulation (Lübbers et al., 2018), cell migration (Bassagañas et al., 2014), chemokine sensing (Kiermaier et al., 2016), and growth factor retention (Sato and Kitajima, 2019).

Lower levels of sialylated gangliosides have been reported in the CNS of elderly individuals (Segler Stahl et al., 1983). In particular, the content of ganglioside-bound Sia in the whole brain of individuals aged 25 to 85 years was found to be reduced by approximately 65% at 85 years of age (Segler Stahl et al., 1983). Furthermore, an increased activity of the endogenous neuraminidases Neu1 and Neu4 was observed during inflammation, which can cleave Sias from the cell surface, suggesting that the Sia cap of the glycocalyx is compromised under pathological inflammatory conditions (Demina et al., 2018; Howlader et al., 2022). Oxidative damage has also been shown to desialylate cell surfaces (Eguchi et al., 2005; Cho et al., 2017). Furthermore, enzymatic desialylation can lead to complement-dependent removal of neurons *in vitro* (Linnartz et al., 2012; Linnartz-Gerlach et al., 2015). Interestingly, oral supplementation with Sia ameliorated the oxidative damage in two different mouse models: a nephropathy model induced by aminoglycoside antibiotics and an oxidative stress model induced by a high-fat diet (Pawluczyk et al., 2015; Yida et al., 2015).

In mice that are heterozygous for the null mutant of UDP-N-acetylglucosamine 2-epimerase/N-acetylmannosamine kinase (*Gne+/−*), a crucial enzyme for Sia biosynthesis, a slight reduction in brain sialylation was noted (Klaus et al., 2020). This reduced sialylation not only led to non-inflammatory synapse and neuronal loss but was also linked to reduced microglial ramification and dependent on complement component 3 (C3) (Klaus et al., 2020). In a follow-up study focusing on the mouse retina, the number of bipolar cells in the retina of 9-month-old Gne+/− mice was reduced, which was again associated with inflammatory processes and dependent on C3 (Cuevas-Rios et al., 2024). Additionally, hyposialylation activated key biological pathways in the mouse retina at 9 months of age, including UV response, epithelial-mesenchymal transition (EMT), p53 pathway, angiogenesis, apoptosis, KRAS signaling, IL6/JAK/STAT3 signaling, tumor necrosis factor-alpha (TNF-A) signaling via NFkB, cholesterol homeostasis, and complement (Cuevas-Rios et al., 2024). Interestingly, most of these enriched gene pathways have been directly or indirectly associated with AMD (Table 1).

**Table 1:** Associations between retinal hyposialylation and age-related macular degeneration (AMD) pathogenesis.

Retinal pathways enriched in mice with hyposialylation (Cuevas-Rios et al., 2024)	Retinal pathway contribution to AMD
UV exposure	Major cause of photoaging (Lueck et al., 2012; Sui et al., 2013)
Epithelial-mesenchymal transition	Pathogenesis of subretinal fibrosis (Kimura et al., 2015; Tuo et al., 2015; Shu et al., 2020; Shen et al., 2023)
Angiogenesis	Pathogenesis of choroidal neovascularization in wet AMD (Ferrara and Adamis, 2016; Wolf et al., 2022)
Apoptosis	Mediating RPE cell death, especially in dry AMD (Hanus et al., 2015; Lenin et al., 2023)
KRAS signaling	Promoting chronic inflammation, oxidative stress, RPE senescence, and impaired autophagy through downstream pathways such as JAK/STAT, PI3K/AKT, MAPK, and mTOR (Wang et al., 2019; Zhang et al., 2020; Ghosh et al., 2024)
IL6/JAK/STAT3 signaling	Driving chronic inflammation and establishing a pro-inflammatory feedback loop (Wiciński et al., 2021; Ahmed et al., 2024)
TNF-A signaling via NFkB	Promoting chronic inflammation and RPE cell apoptosis in dry AMD and inducing VEGF expression in wet AMD (Shi et al., 2006; Khan et al., 2021)
Cholesterol homeostasis	Accumulation of cholesterol and lipid deposits (drusen), impairing cholesterol clearance, and activation of inflammatory pathways (Pikuleva and Curcio, 2014; Ban et al., 2018)
Complement	Detailed discussion in Section 2.1.

Furthermore, several enzymes involved in sialylation and glycocalyx formation are associated with AMD (Emilsson et al., 2022). In a proteogenomic study, Emilsson et al. investigated associations between 4,782 human AMD candidate serum proteins and various disease stages of AMD and found that not only complement and inflammation-related factors were enriched in the serum of patients at risk for early or late AMD, but also that the serum sialyltransferase (ST6 N-acetylgalactosaminide *α*-2,6-sialyltransferase 1, ST6GALNAC1) and fucosyltransferase 5 (FUT5) were associated with early AMD (Emilsson et al., 2022). In a recent pilot study involving human retinal tissue, a reduction in Sia level at the Bruch’s membrane/choroid interface in both the macular and peripheral regions of the retina in individuals with AMD (*n* = 3) was observed compared to controls (*n* = 5; Swan et al., 2025).

### 2.1 Sialylation as a protective mechanism against complement activation in the retina

The retina is particularly susceptible to complement-mediated damage due to its high metabolic activity and exposure to light-induced oxidative stress. The complement system consists of three activation pathways: classical, lectin, and alternative, all of which converge in C3 activation and lead to the formation of immunostimulatory factors such as C3a, C5a, and membrane attack complexes (MAC, C5b-9; Ricklin et al., 2010; Merle et al., 2015). As mentioned above, the loss of rod bipolar cells and the increase in inflammatory gene transcription were prevented after crossing *Gne+/−* mice that show hyposialylation with complement factor C3-deficient animals (Cuevas-Rios et al., 2024).

**Figure 1:**
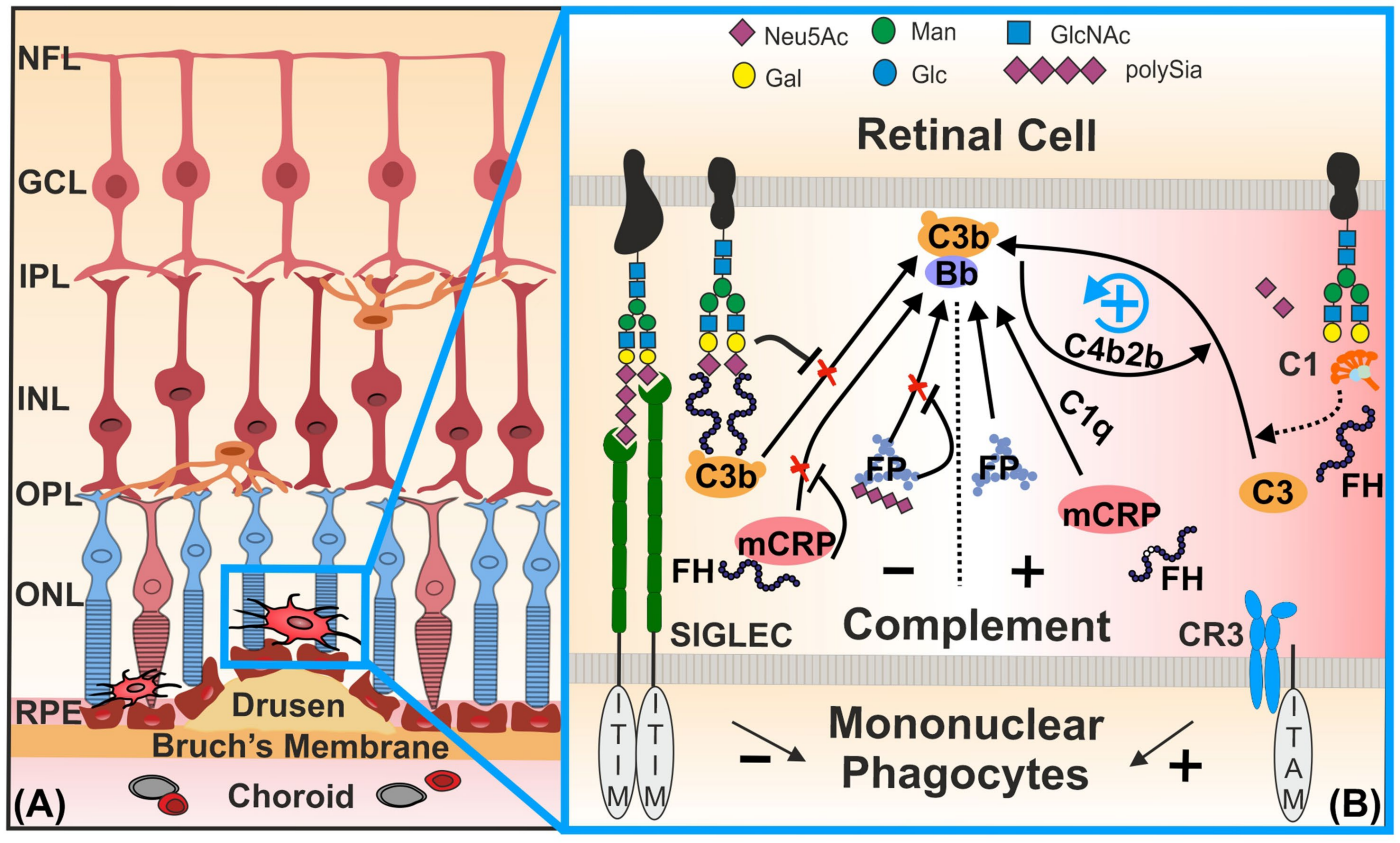
Homeostatic control of the complement system and mononuclear phagocytes by sialylation. **(A)** Mononuclear phagocytes, such as microglia and invaded macrophages, are activated near drusen in inflammatory retinal diseases [e.g., in age-related macular degeneration (AMD)]. **(B)** Sialic acid/N-acetylneuraminic acid (Sia /NeuAc) or polysialic acid (polySia) displayed on glycoproteins [e.g., neural cell adhesion molecule (NCAM) and CD59] and glycolipids (e.g., gangliosides) of the intact glycocalyx engage SIGLEC receptors. Inhibitory retinal microglial SIGLECs—SIGLEC-7, −9, and −11 of humans—possess inhibitory signaling ITIM domains. Ligand binding triggers ITIM phosphorylation, enabling recruitment of phosphatases SHP1/2. This phosphatase activity counteracts signaling cascades initiated by ITAM-associated transmembrane molecules (e.g., TYROBP) that transmit inducing signals of the complement factor CR3. Complement factor H (FH) binds to Sia moieties on the intact host cell glycocalyx (e.g., PTX3), thereby inhibiting the initiation of the C3-mediated alternative complement pathway and blocking assembly of the C3 convertase (C3bBb). Thus, FH actively prevents aberrant activation of the alternative complement cascade on healthy sialylated cells. In contrast, desialylated glycomolecules [e.g., Pentraxin-3 (PTX3)] are opsonized by C1, initiating the classical complement cascade and generating the classical C3 convertase (C4b2b), as indicated by the dashed arrow. The classical C3 convertase then cleaves C3 into C3b and C3a. When C3b binds weakly to the altered cells in the absence of FH binding, it can initiate and amplify the alternative complement pathway via C3bBb. FH also binds to mCRP and thereby prevents its potential to recruit C1q to the surface of damaged cells. Binding of FP to desialylated surfaces drives C3b deposition. It stabilizes the formation and activation of the alternative C3bBb, together with the complement activator properdin (C3bBbP). However, soluble polySia has been shown to reduce the activation of the alternative complement pathway via binding to the FP. In summary, loss or reduction of sialylation—due to neuraminidase activity, oxidative damage, or aging—triggers both classical and alternative complement activation, enhancing C3b deposition and the formation of C3 convertases from both pathways. CR3, complement receptor 3; FH, complement factor H; FP, complement factor properdin; GCL, ganglion cell layer; INL, inner nuclear layer; IPL, inner plexiform layer; ITAM, immunoreceptor tyrosine-based activation motif; ITIM, immunoreceptor tyrosine-based inhibition motif; mCRP, monomeric C-reactive protein; NFL, nerve fiber layer; ONL, outer nuclear layer; OPL, outer plexiform layer; RPE, retinal pigment epithelium; SIGLEC, sialic acid-binding immunoglobulin-like lectins. Figure was created using CorelDraw 2019 (Corel Corporation, Ottawa, Canada).

Complement factor H is a key regulatory protein of the alternative pathway that prevents complement overactivation by inhibiting the formation and accelerating the decay of C3 convertase (Chen et al., 2007; Kopp et al., 2012). The human FH family consists of seven highly related members, namely, FH, FH-like 1 (FHL-1), and FH-related proteins 1 to 5 (FHR-1-5). All family members are composed of repetitive units, short consensus repeats (SCRs), of ~60 amino acids, which are also termed control complement protein domains. The regulatory function of FH depends on the binding to certain polyanions, such as Sia and glycosaminoglycans (Meri and Pangburn, 1990; Clark et al., 2013). The FH at position SCR20 interacts with α2,3-conjugated Sia moieties that are critical for the discrimination of self and non-self surfaces. The binding of FH to these α2,3-conjugated Sia residues on the cytosolic glycoconjugate complex can form a C3b-FH-Sia complex, which inhibits alternative complement pathway initiation and amplification (Figure 1; Blaum et al., 2014). However, studies have shown that the rs1061170 polymorphism (Tyr402His) in the FH gene, which is closely associated with an increased risk of AMD, does not directly affect the known Sia binding domain SCR20 but instead reduces the ability of FH to bind to retinal components, such as lipid peroxides and C-reactive protein (CRP; Molins et al., 2016; Romero-Vazquez et al., 2021; Giralt et al., 2024). Data indicate that intermediate AMD is associated with decreased serum FH levels and an increased serum monomeric C-reactive protein (mCRP)/FH ratio (Figure 1; Giralt et al., 2024). These changes lead to reduced FH levels and decreased protection of sialylated retinal cell surface membranes against complement attack, as well as increased mCRP-mediated activation of downstream complement pathways (Romero-Vázquez et al., 2020).

Pentraxin-3 (PTX3) has been recently proposed to act as an anchoring site for FH in Bruch’s membrane and RPE, where it limits complement-dependent inflammatory response in a mouse model of oxidative stress-induced AMD (Wang et al., 2016). PTX3 is sialylated and is known to bind FH at SCR19-20, in addition to the side SCR7 (Deban et al., 2008; Inforzato et al., 2013). Interestingly, desialylation of PTX3 allows the binding of C1q and activation of the classical complement pathway (Inforzato et al., 2006).

After reduction or loss of Sia, the underlying glycan structures can trigger classical complement pathway activation (Figure 1). Thus, the remaining desialylated glycan cell surface structures are recognized by the complement complex C1 (C1qC1r_2_C1s), triggering a complement cascade response via opsonization and formation of a C4b2b complex, the classical pathway C3 convertase (Linnartz et al., 2012).

It is worth noting that FP plays an opposite function to FH. FP is the only positive regulator of the alternative pathway and prevents the rapid decay of C3/C5 convertases, thereby amplifying complement activity (Figure 1; Kouser et al., 2013; Chen et al., 2018). In an *in vitro* study, it was found that low molecular weight polySia reduced the binding of serum-derived FP to the cell surface of lesioned Hepa-1c1c7 and PC-12 neuroblastoma cells. In addition, polySia can lead to reduced cell lysis and reduced formation of membrane attack complexes (Karlstetter et al., 2016). Furthermore, the addition of polySia to human serum reduced the activity of the alternative complement pathway, which was triggered by plate-bound lipopolysaccharides (LPS) (Shahraz et al., 2022). Thus, polySia found on the glycocalyx attached to the retinal protein neural cell adhesion molecule (NCAM) could also prevent cell membrane damage by reducing the activity of the alternative complement pathway. Importantly, this inhibitory effect was chain length-dependent, since our group recently showed that soluble monosialic or oligosialic acids failed to bind to the complement regulatory protein FH and FP at physiological concentrations (Shahraz et al., 2022).

In summary, these findings highlight the critical role of multivariant recognition of Sia residues of the cell membrane glycocalyx in maintaining homeostasis of the complement system in the retina.

### 2.2 Sialylation regulates mononuclear phagocyte homeostasis via inhibitory SIGLEC receptors

Mononuclear phagocytes include resident microglia, parenchymal macrophages, and circulating monocytes (Wieghofer et al., 2021). Microglia are uniformly distributed on the plexiform layers in the mature retina and thoroughly cover the retinal environment through their dynamically moving branching processes (Damani et al., 2011; Silverman and Wong, 2018). In response to tissue damage, the microglia are activated, and their morphology changes from ramified to amoeboid, enabling them to rapidly migrate to the site of distress and phagocytose pathogens, dead neurons, and cellular debris (Sappington et al., 2022; Brown, 2023; Ronning et al., 2025). However, this activation of immune cells is also a double-edged sword. It has been shown that activated microglia and macrophages accumulate around drusen, leading to the death of photoreceptor cells and RPE cells (Killingsworth et al., 1990; Dietrich et al., 2020). In addition, mononuclear phagocytes accumulating in the subretinal space secrete VEGF, platelet-derived growth factor-*β*, fibroblast growth factor-1, fibroblast growth factor-2, and transforming growth factor-β1 to promote CNV (Li et al., 2017).

Sialylation regulates the mononuclear phagocytes through signaling via their SIGLECs (Linnartz-Gerlach et al., 2014; Thiesler and Hildebrandt, 2024). When sialylation functions properly, microglia remain in a homeostatic state, enabling them to selectively and silently prune misconnected neuronal dendrites (Azevedo et al., 2020; Paolicelli et al., 2022). After transient transition of microglia into an activated state to eliminate damaged or apoptotic cells, SIGLEC receptors help to resolve the inflammatory reaction, thereby preventing ongoing damage to healthy tissues (Paolicelli et al., 2022). This regulatory capacity of SIGLECs is due to the presence of their immunoreceptor tyrosine-based inhibition motifs (ITIMs) and/or ITIM-like motifs in their intracellular signaling tails (Figure 1). Transmembrane proteins with this motif in the cytoplasmic domain could have inhibitory effects by recruiting serine/threonine phosphatase tyrosine-specific Src homology-2 domain-containing phosphatase-1 (SHP-1/PTPN6) or Src homology-2 domain-containing phosphatase-2 (SHP-2/PTPN11; Linnartz-Gerlach et al., 2014; Lünemann et al., 2021). These proteins counter-regulate other immunoreceptors harboring immunoreceptor tyrosine activation motifs (ITAMs) so that immune cell-mediated activation processes, for example, phagocytosis, oxidative burst, and inflammation, are attenuated (Crocker et al., 2007). *In vitro*, Siglec-E of mice recognizes *α*2,3- and *α*2,8-linked Sia residues of the glycocalyx. Upon stimulation with neural debris, this recognition suppresses the phagocytosis of neural debris, downregulates the transcription of pro-inflammatory cytokine genes, and reduces the production of reactive oxygen species (Claude et al., 2013). Moreover, human SIGLEC-11 binds to α2,8-conjugated Sias of the glycocalyx and inhibits LPS-induced gene transcription of pro-inflammatory mediators in cultured mouse microglia ectopically expressing the human SIGLEC-11 receptor, thus demonstrating a neuroprotective function of this human-specific microglial SIGLEC-11 receptor (Wang and Neumann, 2010). Expression of SIGLEC-7, −9, and −11 in retinal tissue from AMD patients was found to be significantly upregulated compared with healthy controls (Krishnan et al., 2023). Interestingly, SIGLEC-7 and −9 of mononuclear phagocytes recognized Sia residues on the sialylated complement inhibitory glycoprotein CD59, thereby suppressing their immune cell attack capacity (Wen et al., 2024).

Thus, ITIM-containing SIGLECs could be a novel therapeutic target for treating inflammatory retinal diseases involving activated mononuclear phagocytes.

### 2.3 Therapeutic potential of polysialylated ligands in age-related retinal diseases

Polysialic acid is an extended homopolymer of α2,8-conjugated Sia that is attached to several glycoproteins, such as neural cell adhesion molecule (NCAM), synCAM-1, neuropilin −2 (NRP2), and CD36, on the surface of mammalian neurons and immune cells (Sato and Kitajima, 2013). In a mouse model of laser-induced retinal injury, intravitreal application of polySia avDP20 reduced the mononuclear phagocyte activation, vascular leakage, and membrane attack complex deposition in humanized SIGLEC-11 transgenic mice (Karlstetter et al., 2016). In addition, *in vitro* studies showed that polySia avDP20 inhibited mononuclear phagocyte reactivity via the SIGLEC receptor and directly interfered with the activation of the alternative complement system (Karlstetter et al., 2016).

Polysialic acid-functionalized nanoparticles (polySia-NPs), in which polySia was conjugated to a core of polyethylene glycol and poly lactic-co-glycolic acid (PLGA) copolymers, were effective in reducing macrophage-induced inflammatory retinal responses after intravitreal application in humanized SIGLEC-11 transgenic mice (Krishnan et al., 2023). In a mouse model of bright light retinal damage, polySia-NPs bound to SIGLEC-E receptors on macrophages and elicited an anti-inflammatory effect with a reduction in IL-6, IL-1β, and TNF-α levels, as well as diminished loss of retinal outer nuclear layer thickness (Krishnan et al., 2023).

The proven *in vivo* safety and efficacy of polySia-NPs in treating animal models of AMD led to an investigational new drug (IND) application at the US Food and Drug Administration (FDA) and received approval for entry into human Phase II/III clinical trials for the treatment of geographic atrophy in AMD patients (Krishnan et al., 2024; SIGLEC study ClinicalTrials.gov Identifier NCT05839041).

## 3 Conclusion

Sialylation plays a critical role in maintaining retinal immune homeostasis and protecting against complement-mediated damage. Age-related hyposialylation disrupts this protective process, contributing to the pathogenesis of retinal diseases, such as AMD. The interplay between hyposialylation and complement activation creates a vicious cycle of chronic inflammation and degeneration, highlighting the need for therapies that target both processes simultaneously. Sia-based polymers, such as polySia, therefore represent a promising therapeutic approach for complement-related retinal diseases.

## References

[ref1] Ahmed C. M. Johnson H. M. Lewin A. S. (2024). Corneal application of SOCS1/3 peptides for the treatment of eye diseases mediated by inflammation and oxidative stress. Front. Immunol. 15:1416181. doi: 10.3389/FIMMU.2024.1416181, PMID: 39104531 PMC11298391

[ref2] Amirul Islam F. M. Chong E. W. Hodge A. M. Guymer R. H. Aung K. Z. Makeyeva G. A.. (2014). Dietary patterns and their associations with age-related macular degeneration: the Melbourne collaborative cohort study. Ophthalmology 121, 1428–1434.e2. doi: 10.1016/J.OPHTHA.2014.01.002, PMID: 24560564

[ref3] Azevedo M. D. Sander S. Tenenbaum L. (2020). GDNF, a neuron-derived factor upregulated in glial cells during disease. J. Clin. Med. 9:456. doi: 10.3390/JCM9020456, PMID: 32046031 PMC7073520

[ref4] Ban N. Lee T. J. Sene A. Choudhary M. Lekwuwa M. Dong Z.. (2018). Impaired monocyte cholesterol clearance initiates age-related retinal degeneration and vision loss. JCI Insight 3:120824. doi: 10.1172/JCI.INSIGHT.120824, PMID: 30185655 PMC6171801

[ref5] Bassagañas S. Pérez-Garay M. Peracaula R. (2014). Cell surface sialic acid modulates extracellular matrix adhesion and migration in pancreatic adenocarcinoma cells. Pancreas 43, 109–117. doi: 10.1097/MPA.0B013E31829D9090, PMID: 23921962

[ref6] Blaum B. S. Hannan J. P. Herbert A. P. Kavanagh D. Uhrín D. Stehle T. (2014). Structural basis for sialic acid–mediated self-recognition by complement factor H. Nat. Chem. Biol. 11, 77–82. doi: 10.1038/nchembio.1696 25402769

[ref7] Brown G. C. (2023). Cell death by phagocytosis. Nat. Rev. Immunol. 24, 91–102. doi: 10.1038/s41577-023-00921-6 37604896

[ref8] Chen J. Y. Cortes C. Ferreira V. P. (2018). Properdin: a multifaceted molecule involved in inflammation and diseases. Mol. Immunol. 102, 58–72. doi: 10.1016/J.MOLIMM.2018.05.018, PMID: 29954621 PMC7375857

[ref9] Chen M. Forrester J. V. Xu H. (2007). Synthesis of complement factor H by retinal pigment epithelial cells is down-regulated by oxidized photoreceptor outer segments. Exp. Eye Res. 84, 635–645. doi: 10.1016/J.EXER.2006.11.015, PMID: 17292886

[ref10] Chen M. Luo C. Zhao J. Devarajan G. Xu H. (2019). Immune regulation in the aging retina. Prog. Retin. Eye Res. 69, 159–172. doi: 10.1016/j.preteyeres.2018.10.003, PMID: 30352305 PMC6373845

[ref11] Cho A. Christine M. Malicdan V. Miyakawa M. Nonaka I. Nishino I.. (2017). Sialic acid deficiency is associated with oxidative stress leading to muscle atrophy and weakness in GNE myopathy. Hum. Mol. Genet. 26, 3081–3093. doi: 10.1093/HMG/DDX192, PMID: 28505249 PMC6075185

[ref12] Chou H. H. Hayakawa T. Diaz S. Krings M. Indriati E. Leakey M.. (2002). Inactivation of CMP-N-acetylneuraminic acid hydroxylase occurred prior to brain expansion during human evolution. Proc. Natl. Acad. Sci. USA 99, 11736–11741. doi: 10.1073/PNAS.182257399, PMID: 12192086 PMC129338

[ref13] Clark S. J. Ridge L. A. Herbert A. P. Hakobyan S. Mulloy B. Lennon R.. (2013). Tissue-specific host recognition by complement factor H is mediated by differential activities of its glycosaminoglycan-binding regions. J. Immunol. 190, 2049–2057. doi: 10.4049/JIMMUNOL.1201751, PMID: 23365078 PMC3672945

[ref14] Claude J. Linnartz-Gerlach B. Kudin A. P. Kunz W. S. Neumann H. (2013). Microglial CD33-related Siglec-E inhibits neurotoxicity by preventing the phagocytosis-associated oxidative burst. J. Neurosci. 33, 18270–18276. doi: 10.1523/JNEUROSCI.2211-13.2013, PMID: 24227736 PMC3828472

[ref15] Crocker P. R. Paulson J. C. Varki A. (2007). Siglecs and their roles in the immune system. Nat. Rev. Immunol. 7, 255–266. doi: 10.1038/nri2056 17380156

[ref16] Cuevas-Rios G. Assale T. A. Wissfeld J. Bungartz A. Hofmann J. Langmann T.. (2024). Decreased sialylation elicits complement-related microglia response and bipolar cell loss in the mouse retina. Glia 72, 2295–2312. doi: 10.1002/GLIA.24613, PMID: 39228105 PMC13058866

[ref17] Damani M. R. Zhao L. Fontainhas A. M. Amaral J. Fariss R. N. Wong W. T. (2011). Age-related alterations in the dynamic behavior of microglia. Aging Cell 10, 263–276. doi: 10.1111/J.1474-9726.2010.00660.X, PMID: 21108733 PMC3056927

[ref18] Deban L. Jarva H. Lehtinen M. J. Bottazzi B. Bastone A. Doni A.. (2008). Binding of the long Pentraxin PTX3 to factor H: interacting domains and function in the regulation of complement activation. J. Immunol. 181, 8433–8440. doi: 10.4049/JIMMUNOL.181.12.8433, PMID: 19050261

[ref19] Demina E. P. Pierre W. C. Nguyen A. L. A. Londono I. Reiz B. Zou C.. (2018). Persistent reduction in sialylation of cerebral glycoproteins following postnatal inflammatory exposure. J. Neuroinflammation 15, 1–14. doi: 10.1186/S12974-018-1367-2 30518374 PMC6282350

[ref20] Dietrich L. Lucius R. Roider J. Klettner A. (2020). Interaction of inflammatorily activated retinal pigment epithelium with retinal microglia and neuronal cells. Exp. Eye Res. 199:108167. doi: 10.1016/J.EXER.2020.108167, PMID: 32735798

[ref21] Eguchi H. Ikeda Y. Ookawara T. Koyota S. Fujiwara N. Honke K.. (2005). Modification of oligosaccharides by reactive oxygen species decreases sialyl Lewis x-mediated cell adhesion. Glycobiology 15, 1094–1101. doi: 10.1093/GLYCOB/CWJ003, PMID: 16000697

[ref22] Emilsson V. Gudmundsson E. F. Jonmundsson T. Jonsson B. G. Twarog M. Gudmundsdottir V.. (2022). A proteogenomic signature of age-related macular degeneration in blood. Nat. Commun. 13, 1–15. doi: 10.1038/s41467-022-31085-x, PMID: 35697682 PMC9192739

[ref23] Ferrara N. Adamis A. P. (2016). Ten years of anti-vascular endothelial growth factor therapy. Nat. Rev. Drug Discov. 15, 385–403. doi: 10.1038/nrd.2015.17 26775688

[ref24] Ghosh S. Hose S. Sinha D. (2024). AKT2-mediated lysosomal dysfunction promotes secretory autophagy in retinal pigment epithelium (RPE) cells. Autophagy 20, 2841–2842. doi: 10.1080/15548627.2024.2413305, PMID: 39412071 PMC11587834

[ref25] Giralt L. Figueras-Roca M. Eguileor B. D. L. Romero B. Zarranz-Ventura J. Alforja S.. (2024). C-reactive protein-complement factor H axis as a biomarker of activity in early and intermediate age-related macular degeneration. Front. Immunol. 15:1330913. doi: 10.3389/FIMMU.2024.1330913 38633250 PMC11021604

[ref26] Hanus J. Anderson C. Wang S. (2015). RPE necroptosis in response to oxidative stress and in AMD. Ageing Res. Rev. 24, 286–298. doi: 10.1016/J.ARR.2015.09.002, PMID: 26369358 PMC4661094

[ref27] Hedlund M. Padler-Karavani V. Varki N. M. Varki A. (2008). Evidence for a human-specific mechanism for diet and antibody-mediated inflammation in carcinoma progression. Proc. Natl. Acad. Sci. USA 105, 18936–18941. doi: 10.1073/PNAS.0803943105, PMID: 19017806 PMC2596253

[ref28] Howlader M. A. Demina E. P. Samarani S. Guo T. Caillon A. Ahmad A.. (2022). The Janus-like role of neuraminidase isoenzymes in inflammation. FASEB J. 36:e22285. doi: 10.1096/FJ.202101218R, PMID: 35363389 PMC9323473

[ref29] Inforzato A. Peri G. Doni A. Garlanda C. Mantovani A. Bastone A.. (2006). Structure and function of the long pentraxin PTX3 glycosidic moiety: fine-tuning of the interaction with C1q and complement activation. Biochemistry 45, 11540–11551. doi: 10.1021/BI0607453, PMID: 16981714

[ref30] Inforzato A. Reading P. C. Barbati E. Bottazzi B. Garlanda C. Mantovani A. (2013). The “sweet” side of a long pentraxin: how glycosylation affects PTX3 functions in innate immunity and inflammation. Front. Immunol. 3, 371–315. doi: 10.3389/FIMMU.2012.00407, PMID: 23316195 PMC3539679

[ref31] Karlstetter M. Kopatz J. Aslanidis A. Shahraz A. Caramoy A. Linnartz-Gerlach B.. (2016). Polysialic acid blocks mononuclear phagocyte reactivity, inhibits complement activation, and protects from vascular damage in the retina. EMBO Mol. Med. 9:154. doi: 10.15252/EMMM.201606627, PMID: 28003336 PMC5286381

[ref32] Kelm S. Schauer R. Manuguerra J. C. Gross H. J. Crocker P. R. (1994). Modifications of cell surface sialic acids modulate cell adhesion mediated by sialoadhesin and CD22. Glycoconj. J. 11, 576–585. doi: 10.1007/BF00731309, PMID: 7696861

[ref33] Khan A. H. Pierce C. O. De Salvo G. Griffiths H. Nelson M. Cree A. J.. (2021). The effect of systemic levels of TNF-alpha and complement pathway activity on outcomes of VEGF inhibition in neovascular AMD. Eye 36, 2192–2199. doi: 10.1038/S41433-021-01824-3, PMID: 34750590 PMC9581945

[ref34] Kiermaier E. Moussion C. Veldkamp C. T. Gerardy-Schahn R. De Vries I. Williams L. G.. (2016). Polysialylation controls dendritic cell trafficking by regulating chemokine recognition. Science 351, 186–190. doi: 10.1126/SCIENCE.AAD0512, PMID: 26657283 PMC5583642

[ref35] Killingsworth M. C. Sarks J. P. Sarks S. H. (1990). Macrophages related to Bruch’s membrane in age-related macular degeneration. Eye 4, 613–621. doi: 10.1038/eye.1990.86 2226993

[ref36] Kimura K. Orita T. Liu Y. Yang Y. Tokuda K. Kurakazu T.. (2015). Attenuation of EMT in RPE cells and subretinal fibrosis by an RAR-γ agonist. J. Mol. Med. (Berl) 93, 749–758. doi: 10.1007/S00109-015-1289-8, PMID: 25947075

[ref37] Klaus C. Hansen J. N. Ginolhac A. Gérard D. Gnanapragassam V. S. Horstkorte R.. (2020). Reduced sialylation triggers homeostatic synapse and neuronal loss in middle-aged mice. Neurobiol. Aging 88, 91–107. doi: 10.1016/J.NEUROBIOLAGING.2020.01.008, PMID: 32087947

[ref38] Klaus C. Liao H. Allendorf D. H. Brown G. C. Neumann H. (2021). Sialylation acts as a checkpoint for innate immune responses in the central nervous system. Glia 69, 1619–1636. doi: 10.1002/GLIA.23945, PMID: 33340149

[ref39] Kopp A. Hebecker M. Svobodová E. Józsi M. (2012). Factor H: a complement regulator in health and disease, and a mediator of cellular interactions. Biomol. Ther. 2, 46–75. doi: 10.3390/BIOM2010046, PMID: 24970127 PMC4030870

[ref40] Kouser L. Abdul-Aziz M. Nayak A. Stover C. M. Sim R. B. Kishore U. (2013). Properdin and factor H: opposing players on the alternative complement pathway “see-saw”. Front. Immunol. 4:47548. doi: 10.3389/FIMMU.2013.00093 PMC3632793 23630525

[ref41] Krishnan A. Callanan D. G. Sendra V. G. Lad A. Christian S. Earla R.. (2024). Comprehensive ocular and systemic safety evaluation of polysialic acid-decorated immune modulating therapeutic nanoparticles (PolySia-NPs) to support entry into first-in-human clinical trials. Pharmaceuticals 17:481. doi: 10.3390/PH17040481 38675441 PMC11054942

[ref42] Krishnan A. Sendra V. G. Patel D. Lad A. Greene M. K. Smyth P.. (2023). Polysialic acid-nanoparticles inhibit macrophage mediated inflammation through Siglec agonism: a potential treatment for age related macular degeneration. Front. Immunol. 14:1237016. doi: 10.3389/FIMMU.2023.1237016 38045700 PMC10690618

[ref43] Lenin R. R. Koh Y. H. Zhang Z. Yeo Y. Z. Parikh B. H. Seah I.. (2023). Dysfunctional autophagy, Proteostasis, and mitochondria as a prelude to age-related macular degeneration. Int. J. Mol. Sci. 24:8763. doi: 10.3390/IJMS24108763, PMID: 37240109 PMC10217992

[ref44] Li L. Heiduschka P. Alex A. F. Niekämper D. Eter N. (2017). Behaviour of CD11b-positive cells in an animal model of laser-induced choroidal neovascularisation. Ophthalmologica 237, 29–41. doi: 10.1159/000453550, PMID: 28092911

[ref45] Linnartz B. Kopatz J. Tenner A. J. Neumann H. (2012). Sialic acid on the neuronal Glycocalyx prevents complement C1 binding and complement Receptor-3-mediated removal by microglia. J. Neurosci. 32, 946–952. doi: 10.1523/JNEUROSCI.3830-11.2012, PMID: 22262892 PMC4037907

[ref46] Linnartz-Gerlach B. Mathews M. Neumann H. (2014). Sensing the neuronal glycocalyx by glial sialic acid binding immunoglobulin-like lectins. Neuroscience 275, 113–124. doi: 10.1016/J.NEUROSCIENCE.2014.05.061, PMID: 24924144

[ref47] Linnartz-Gerlach B. Schuy C. Shahraz A. Tenner A. J. Neumann H. (2015). Sialylation of neurites inhibits complement-mediated macrophage removal in a human macrophage-neuron co-culture system. Glia 64:35. doi: 10.1002/GLIA.22901, PMID: 26257016 PMC4715670

[ref48] Lübbers J. Rodríguez E. van Kooyk Y. (2018). Modulation of immune tolerance via Siglec-sialic acid interactions. Front. Immunol. 9:422533. doi: 10.3389/FIMMU.2018.02807, PMID: 30581432 PMC6293876

[ref49] Lueck K. Hennig M. Lommatzsch A. Pauleikhoff D. Wasmuth S. (2012). Complement and UV-irradiated photoreceptor outer segments increase the cytokine secretion by retinal pigment epithelial cells. Invest. Ophthalmol. Vis. Sci. 53, 1406–1413. doi: 10.1167/IOVS.11-8889, PMID: 22323489

[ref50] Lünemann J. D. von Gunten S. Neumann H. (2021). Targeting sialylation to treat central nervous system diseases. Trends Pharmacol. Sci. 42, 998–1008. doi: 10.1016/J.TIPS.2021.09.002, PMID: 34607695

[ref51] Marchesi N. Capierri M. Pascale A. Barbieri A. (2024). Different therapeutic approaches for dry and wet AMD. Int. J. Mol. Sci. 25:13053. doi: 10.3390/IJMS252313053, PMID: 39684764 PMC11641571

[ref52] Martin M. J. Rayner J. C. Gagneux P. Barnwell J. W. Varki A. (2005). Evolution of human-chimpanzee differences in malaria susceptibility: relationship to human genetic loss of N-glycolylneuraminic acid. Proc. Natl. Acad. Sci. USA 102, 12819–12824. doi: 10.1073/PNAS.0503819102, PMID: 16126901 PMC1200275

[ref53] Meri S. Pangburn M. K. (1990). Discrimination between activators and nonactivators of the alternative pathway of complement: regulation via a sialic acid/polyanion binding site on factor H. Proc. Natl. Acad. Sci. USA 87, 3982–3986. 1692629 10.1073/pnas.87.10.3982 PMC54028

[ref54] Merle N. S. Noe R. Halbwachs-Mecarelli L. Fremeaux-Bacchi V. Roumenina L. T. (2015). Complement system part II: role in immunity. Front. Immunol. 6:136998. doi: 10.3389/FIMMU.2015.00257 PMC4443744 26074922

[ref55] Mitchell P. Liew G. Gopinath B. Wong T. Y. (2018). Age-related macular degeneration. Lancet 392, 1147–1159. doi: 10.1016/S0140-6736(18)31550-2, PMID: 30303083

[ref56] Möckl L. Horst A. K. Kolbe K. Lindhorst T. K. Bräuchle C. (2015). Microdomain formation controls spatiotemporal dynamics of cell-surface glycoproteins. Chembiochem 16, 2023–2028. doi: 10.1002/CBIC.201500361, PMID: 26296625

[ref57] Molins B. Fuentes-Prior P. Adán A. Antón R. Arostegui J. I. Yagüe J.. (2016). Complement factor H binding of monomeric C-reactive protein downregulates proinflammatory activity and is impaired with at risk polymorphic CFH variants. Sci. Rep. 6, 1–12. doi: 10.1038/srep22889, PMID: 26961257 PMC4785391

[ref58] Murakami Y. Ishikawa K. Nakao S. Sonoda K. H. (2020). Innate immune response in retinal homeostasis and inflammatory disorders. Prog. Retin. Eye Res. 74:100778. doi: 10.1016/J.PRETEYERES.2019.100778, PMID: 31505218

[ref59] Nycholat C. M. Rademacher C. Kawasaki N. Paulson J. C. (2012). In silico-aided design of a glycan ligand of sialoadhesin for in vivo targeting of macrophages. J. Am. Chem. Soc. 134, 15696–15699. doi: 10.1021/JA307501E, PMID: 22967315 PMC3462166

[ref60] Oetke C. Hinderlich S. Brossmer R. Reutter W. Pawlita M. Keppler O. T. (2001). Evidence for efficient uptake and incorporation of sialic acid by eukaryotic cells. Eur. J. Biochem. 268, 4553–4561. doi: 10.1046/J.1432-1327.2001.02379.X, PMID: 11502217

[ref61] Paolicelli R. C. Sierra A. Stevens B. Tremblay M. E. Aguzzi A. Ajami B.. (2022). Microglia states and nomenclature: a field at its crossroads. Neuron 110, 3458–3483. doi: 10.1016/J.NEURON.2022.10.020, PMID: 36327895 PMC9999291

[ref62] Pawluczyk I. Z. A. Najafabadi M. G. Brown J. R. Bevington A. Topham P. S. (2015). Sialic acid supplementation ameliorates puromycin aminonucleoside nephrosis in rats. Lab. Investig. 95, 1019–1028. doi: 10.1038/LABINVEST.2015.78, PMID: 26121320

[ref63] Pearce O. M. T. Läubli H. (2016). Sialic acids in cancer biology and immunity. Glycobiology 26, 111–128. doi: 10.1093/GLYCOB/CWV097, PMID: 26518624

[ref64] Pikuleva I. A. Curcio C. A. (2014). Cholesterol in the retina: the best is yet to come. Prog. Retin. Eye Res. 41, 64–89. doi: 10.1016/J.PRETEYERES.2014.03.002, PMID: 24704580 PMC4058366

[ref65] Ricklin D. Hajishengallis G. Yang K. Lambris J. D. (2010). Complement: a key system for immune surveillance and homeostasis. Nat. Immunol. 11, 785–797. doi: 10.1038/ni.1923, PMID: 20720586 PMC2924908

[ref66] Rillahan C. D. Schwartz E. McBride R. Fokin V. V. Paulson J. C. (2012). ‘Click and pick’ identification of high affinity Sialoside ligands for Siglec-based targeting of leukocytes. Angew. Chem. Int. Ed. Eng. 51:11014. doi: 10.1002/ANIE.201205831 PMC3480989 23038623

[ref67] Romero-Vázquez S. Adán A. Figueras-Roca M. Llorenç V. Slevin M. Vilahur G.. (2020). Activation of C-reactive protein proinflammatory phenotype in the blood retinal barrier in vitro: implications for age-related macular degeneration. Aging (Albany NY) 12, 13905–13923. doi: 10.18632/AGING.103655, PMID: 32673285 PMC7425453

[ref68] Romero-vazquez S. Llorens V. Soler-boronat A. Figueras-roca M. Adan A. Molins B. (2021). Interlink between inflammation and oxidative stress in age-related macular degeneration: role of complement factor H. Biomedicine 9:763. doi: 10.3390/BIOMEDICINES9070763, PMID: 34209418 PMC8301356

[ref69] Ronning K. E. Burns M. E. Sennlaub F. (2025). Monocytes in retinal degeneration: little cells with a big impact. Adv. Exp. Med. Biol. 1468, 133–137. doi: 10.1007/978-3-031-76550-6_22, PMID: 39930185

[ref70] Saddala M. S. Lennikov A. Mukwaya A. Fan L. Hu Z. Huang H. (2019). Transcriptome-wide analysis of differentially expressed chemokine receptors, SNPs, and SSRs in the age-related macular degeneration. Hum. Genomics 13:15. doi: 10.1186/S40246-019-0199-1, PMID: 30894217 PMC6425613

[ref71] Sappington R. M. Joachim S. C. Freude K. Guo L. Choi S. Bikkannavar P.. (2022). Microglia: key players in retinal ageing and neurodegeneration. Front. Cell. Neurosci. 16:804782. doi: 10.3389/FNCEL.2022.804782 35370560 PMC8968040

[ref72] Sato C. Kitajima K. (2013). Disialic, oligosialic and polysialic acids: distribution, functions and related disease. J. Biochem. 154, 115–136. doi: 10.1093/JB/MVT057, PMID: 23788662

[ref73] Sato C. Kitajima K. (2019). Sialic acids in neurology. Adv. Carbohydr. Chem. Biochem. 76, 1–64. doi: 10.1016/BS.ACCB.2018.09.003, PMID: 30851742

[ref74] Schnaar R. L. Gerardy-Schahn R. Hildebrandt H. (2014). Sialic acids in the brain: gangliosides and polysialic acid in nervous system development, stability, disease, and regeneration. Physiol. Rev. 94, 461–518. doi: 10.1152/PHYSREV.00033.2013, PMID: 24692354 PMC4044301

[ref75] Segler Stahl K. Webster J. C. Brunngraber E. G. (1983). Changes in the concentration and composition of human brain gangliosides with aging. Gerontology 29, 161–168. 6852543 10.1159/000213109

[ref76] Shahraz A. Lin Y. Mbroh J. Winkler J. Liao H. Lackmann M.. (2022). Low molecular weight polysialic acid binds to properdin and reduces the activity of the alternative complement pathway. Sci. Rep. 12, 1–12. doi: 10.1038/s41598-022-09407-2, PMID: 35388026 PMC8987038

[ref77] Shen G. Li Y. Zeng Y. Hong F. Zhang J. Wang Y.. (2023). Kallistatin deficiency induces the oxidative stress-related epithelial-mesenchymal transition of retinal pigment epithelial cells: a novel protagonist in age-related macular degeneration. Invest. Ophthalmol. Vis. Sci. 64:15. doi: 10.1167/IOVS.64.12.15, PMID: 37682567 PMC10500364

[ref78] Shi X. Semkova I. Müther P. S. Dell S. Kociok N. Joussen A. M. (2006). Inhibition of TNF-alpha reduces laser-induced choroidal neovascularization. Exp. Eye Res. 83, 1325–1334. doi: 10.1016/J.EXER.2006.07.007, PMID: 16959248

[ref79] Shu D. Y. Butcher E. Saint-Geniez M. (2020). EMT and EndMT: emerging roles in age-related macular degeneration. Int. J. Mol. Sci. 21, 1–26. doi: 10.3390/IJMS21124271 PMC7349630 32560057

[ref80] Silverman S. M. Wong W. T. (2018). Microglia in the retina: roles in development, maturity, and disease. Annu Rev Vis Sci 4, 45–77. doi: 10.1146/ANNUREV-VISION-091517-034425, PMID: 29852094

[ref81] Streilein J. W. Ma N. Wenkel H. Fong Ng T. Zamiri P. (2002). Immunobiology and privilege of neuronal retina and pigment epithelium transplants. Vis. Res. 42, 487–495. doi: 10.1016/S0042-6989(01)00185-7, PMID: 11853765

[ref82] Sui G. Y. Liu G. C. Liu G. Y. Gao Y. Y. Deng Y. Wang W. Y.. (2013). Is sunlight exposure a risk factor for age-related macular degeneration? A systematic review and meta-analysis. Br. J. Ophthalmol. 97, 389–394. doi: 10.1136/BJOPHTHALMOL-2012-302281, PMID: 23143904

[ref83] Swan J. Toomey C. B. Bergstrand M. Cuello H. A. Robie J. Yu H.. (2025). The sialome of the retina, alteration in age-related macular degeneration (AMD) pathology and potential impacts on complement factor H. bioRxiv. doi: 10.1101/2025.03.09.642149, PMID: 40576434 PMC12212450

[ref84] Tangvoranuntakul P. Gagneux P. Diaz S. Bardor M. Varki N. Varki A.. (2003). Human uptake and incorporation of an immunogenic nonhuman dietary sialic acid. Proc. Natl. Acad. Sci. USA 100, 12045–12050. doi: 10.1073/PNAS.2131556100, PMID: 14523234 PMC218710

[ref85] Thiesler H. Hildebrandt H. (2024). Polysialic acid-Siglec immune checkpoints of microglia and macrophages: perspectives for therapeutic intervention. Neural Regen. Res. 21, 661–662. doi: 10.4103/NRR.NRR-D-24-01195, PMID: 39688555 PMC12220714

[ref86] Thomas C. J. Mirza R. G. Gill M. K. (2021). Age-related macular degeneration. Med. Clin. North Am. 105, 473–491. doi: 10.1016/J.MCNA.2021.01.003, PMID: 33926642

[ref87] Tuo J. Wang Y. Cheng R. Li Y. Chen M. Qiu F.. (2015). Wnt signaling in age-related macular degeneration: human macular tissue and mouse model. J. Transl. Med. 13:330. doi: 10.1186/S12967-015-0683-X, PMID: 26476672 PMC4609061

[ref88] Varki A. Gagneux P. (2012). Multifarious roles of sialic acids in immunity. Ann. N. Y. Acad. Sci. 1253, 16–36. doi: 10.1111/J.1749-6632.2012.06517.X, PMID: 22524423 PMC3357316

[ref89] Varki A. Schnaar R. L. Schauer R. (2015). Sialic acids and other nonulosonic acids. essentials of glycobiology (3rd ed.). Cold Spring Harbor (NY): Cold Spring Harbor Laboratory Press. 179–195.

[ref90] Wang L. Cano M. Datta S. Wei H. Ebrahimi K. B. Gorashi Y.. (2016). Pentraxin 3 recruits complement factor H to protect against oxidative stress-induced complement and inflammasome overactivation. J. Pathol. 240, 495–506. doi: 10.1002/PATH.4811, PMID: 27659908

[ref91] Wang Y. Neumann H. (2010). Alleviation of neurotoxicity by microglial human Siglec-11. J. Neurosci. 30, 3482–3488. doi: 10.1523/JNEUROSCI.3940-09.2010, PMID: 20203208 PMC6634112

[ref92] Wang S. Wang X. Cheng Y. Ouyang W. Sang X. Liu J.. (2019). Autophagy dysfunction, cellular senescence, and abnormal immune-inflammatory responses in AMD: from mechanisms to therapeutic potential. Oxidative Med. Cell. Longev. 2019:3632169. doi: 10.1155/2019/3632169, PMID: 31249643 PMC6556250

[ref93] Wen R. M. Stark J. C. Marti G. E. W. Fan Z. Lyu A. Marques F. J. G.. (2024). Sialylated glycoproteins suppress immune cell killing by binding to Siglec-7 and Siglec-9 in prostate cancer. J. Clin. Invest. 134:180282. doi: 10.1172/JCI180282 PMC11645153 39436703

[ref94] Wiciński M. Seredyka-Burduk M. Liberski S. Marczak D. Pol M. Malinowski B.. (2021). Evaluation of blood coagulation parameters and ADMA, NO, IL-6, and IL-18 serum levels in patients with Neovascular AMD before, during, and after the initial loading phase of intravitreal Aflibercept. Life (Basel) 11:441. doi: 10.3390/LIFE11050441, PMID: 34069173 PMC8156295

[ref95] Wieghofer P. Hagemeyer N. Sankowski R. Schlecht A. Staszewski O. Amann L.. (2021). Mapping the origin and fate of myeloid cells in distinct compartments of the eye by single-cell profiling. EMBO J. 40:e105123. doi: 10.15252/EMBJ.2020105123, PMID: 33555074 PMC7957431

[ref96] Wolf A. T. Harris A. Oddone F. Siesky B. Verticchio Vercellin A. Ciulla T. A. (2022). Disease progression pathways of wet AMD: opportunities for new target discovery. Expert Opin. Ther. Targets 26, 5–12. doi: 10.1080/14728222.2022.2030706, PMID: 35060431 PMC8915198

[ref97] Yida Z. Imam M. U. Ismail M. Ismail N. Ideris A. Abdullah M. A. (2015). High fat diet-induced inflammation and oxidative stress are attenuated by N-acetylneuraminic acid in rats. J. Biomed. Sci. 22, 1–10. doi: 10.1186/S12929-015-0211-6 26498218 PMC4619312

[ref98] Zhang M. Jiang N. Chu Y. Postnikova O. Varghese R. Horvath A.. (2020). Dysregulated metabolic pathways in age-related macular degeneration. Sci. Rep. 10:2464. doi: 10.1038/S41598-020-59244-4, PMID: 32051464 PMC7016007

[ref99] Zhong X. D’antona A. M. Scarcelli J. J. Rouse J. C. (2022). New opportunities in glycan engineering for therapeutic proteins. Antibodies 11:5. doi: 10.3390/ANTIB11010005, PMID: 35076453 PMC8788452

